# Long‐term clinical and MRI outcomes of a polyurethane meniscal scaffold implantation for the treatment of partial meniscal deficiency: A minimum 10‐year follow‐up study

**DOI:** 10.1002/ksa.12724

**Published:** 2025-06-15

**Authors:** Vasileios S. Akrivos, George A. Komnos, Bela Ujvari, Artemis Hante, Eleftheria Adaktylidou, Theofilos Karachalios, Michael Hantes

**Affiliations:** ^1^ Department of Orthopaedic Surgery and Musculoskeletal Trauma, Faculty of Medicine, School of Health Sciences University of Thessalia Larissa Greece; ^2^ Department of Traumatology University of Semmelweiss Budapest Hungary; ^3^ Department of Physiotherapy International Hellenic University Nea Moudania Greece

**Keywords:** Actifit, irreparable meniscal defect, meniscus, partial meniscectomy, polyurethane scaffold, scaffold

## Abstract

**Purpose:**

To assess the clinical and magnetic resonance imaging (MRI) results after implantation of a polyurethane scaffold for chronic segmental meniscus deficiency following partial meniscectomy in a long‐term follow‐up.

**Study Design:**

Level IV, retrospective case series.

**Methods:**

Thirty‐two knees received arthroscopic implantation of an Actifit® polyurethane meniscal implant for deficiency of the meniscus. Clinical outcomes were assessed using patient‐reported outcome scores, including Knee injury and Osteoarthritis Outcome Score (KOOS), Lysholm, Tegner Activity Scale and visual analogue scale (VAS) for pain and satisfaction. Preoperative MRI scans and final follow‐up radiographs and MRI evaluations were performed to assess scaffold morphology, tissue integration and the status of the articular cartilage.

**Results:**

The mean follow‐up was 11.4 years. Significant improvements were observed in clinical outcomes, with KOOS improving from a preoperative mean of 47.75 (standard deviation [SD]: 16.88) to a 78.62 (SD: 15.45), Lysholm scores from 46.62 (SD: 16.96) to 84.62 (SD: 13.03), Tegner Activity Scale improving from 1.8 to 4.1 at the final follow‐up. The VAS for pain decreased from a preoperative mean of 5.62 (SD: 2.54) to 2.12 (SD: 2.02). MRI evaluations using the Genovese classification showed 23 knees with Type II scaffolds, 0 with Type III and 9 with Type I. Bone oedema was not present in 27 knees, with 5 knees showing Type I bone oedema. Meniscal extrusion was observed in 24 knees with partial extrusion and 8 knees with complete extrusion. The contralateral meniscus was normal in 28 knees and graded as Reicher Grade 1 in 2 knees. The Kellgren–Lawrence classification revealed 15 knees with Type I osteoarthritic changes, 10 with Type II changes and 7 with Type III changes.

**Conclusion:**

Arthroscopic treatment for patients with chronic segmental meniscal loss using a polyurethane meniscal implant can achieve sustainable long‐term results regarding pain reduction and knee function. However, the MRI did not reveal normal menisci in all cases.

**Level of Evidence:**

Level IV.

**Abbreviations**
ACL: anterior cruciate ligament
Actifit®: brand name of the polyurethane meniscal scaffold
CI: confidence interval
IKDC: International Knee Documentation Committee
KOOS: Knee injury and Osteoarthritis Outcome Score
MCID: minimum clinically important difference
MRI: magnetic resonance imaging
SD: standard deviation
VAS: visual analogue scale

## INTRODUCTION

Meniscal tears are among the most common knee injuries, often necessitating surgical intervention. Many meniscal tears are irreparable, and partial meniscectomy is necessary [4, 21]. Each year, approximately 60–70 meniscectomies are performed per 100,000 inhabitants [14]. Alterations in the biomechanical status and homoeostasis of the knee can progressively lead to cartilage degeneration and pain, ultimately resulting in secondary osteoarthritis [9]. This highlights the critical need to explore options for replacing the damaged meniscus.

Actifit, a synthetic polycaprolactone‐polyurethane scaffold, offers a more durable option compared to collagen meniscal implant. It is designed to address partial meniscus defects and is more resistant to surgical procedures and mechanical loads. The polyurethane structure of Actifit supports cellular ingrowth, development of neo vessels and promoting the formation of tissue resembling the native meniscus [5, 20]. While short‐term results indicate significant functional improvement, long‐term studies are needed to fully understand its efficacy in cartilage preservation and overall knee function.

This study aims to evaluate the functional outcomes of Actifit implantation following partial meniscectomy over a minimum follow‐up period of ten years. Additionally, it seeks to assess the condition of the implant and cartilage using radiographs and magnetic resonance imaging (MRI). We hypothesize that polyurethane scaffold implantation provides improvement in pain and knee function, while limiting the progression of degenerative joint changes over the long term. The findings will provide crucial insights into the long‐term viability of Actifit as a meniscal substitute and its potential role in mitigating the degenerative changes associated with partial meniscectomy.

## METHODS

This retrospective study, based on prospectively collected data, included patients who underwent Actifit® polyurethane meniscal implant arthroscopic implantation for meniscal segmental defects. The study spanned from January 2008 to January 2014. All participants provided written informed consent prior to their inclusion in the study. Institutional review board approval was obtained (approval number: 2957).

### Inclusion criteria

The primary inclusion criterion was post‐meniscectomy syndrome accompanied by knee pain in the involved compartment. Patients were eligible if preoperative MRI confirmed partial meniscal loss with an intact peripheral rim, as well as preserved anterior and posterior horns. The age range for inclusion was restricted to 16–40 years. Additionally, patients were required to demonstrate normal joint alignment, defined as a mechanical tibiofemoral angle of ≤2°, exhibit no evidence of arthritic changes or only doubtful joint space narrowing (Kellgren–Lawrence [K–L] Grade 0 or I) on radiographic evaluation and ligamentous stability of the knee.

### Surgical technique for Actifit® polyurethane meniscal implant

The Actifit® polyurethane meniscal implant was implemented using a standardized surgical approach. This approach involved arthroscopic implantation via standard anterolateral and anteromedial portals. The remnants of the meniscus were meticulously trimmed back to the vascular zone to ensure a healthy and well‐vascularized environment for the implant. The defect size was then measured using a specialized ruler, and the scaffold was cut to fit the defect, incorporating a 10% oversizing to account for any potential shrinkage or deformation post‐implantation. The fixation of the implant was achieved through a hybrid suture technique using the combination of all inside and outside‐out sutures to ensure stability of the scaffold.

### Post‐operative rehabilitation

The post‐operative rehabilitation programme emphasized pain management and swelling reduction, with partial weight‐bearing using crutches. Range of motion exercises were focused on knee flexion and extension. Patients wore a hinged knee brace for 6 weeks. In the first week, knee flexion was limited to 30°. From Weeks 2 to 6, flexion increased by 10° per week, aiming to reach 90° of flexion by the end of Week 6.

### Outcome measures

To assess the outcomes of the surgical intervention, both clinical and radiological evaluations were performed. Clinical outcomes were measured using several patient‐reported outcome scores, including the Knee injury and Osteoarthritis Outcome Score (KOOS), Lysholm score, visual analogue scale (VAS) for pain and Tegner Activity Scale and patient satisfaction on a visual scale from 0 to 10. These tools provided a comprehensive evaluation of the patients' pain levels, functional abilities, and overall satisfaction with the procedure. Preoperative MRI scans were used to establish baseline conditions, and follow‐up MRI and X‐ray evaluations were conducted at the final follow‐up visit. Radiographical evaluation was done using a full weight‐bearing standing anteroposterior radiograph. The MRI scans were performed using a 3‐T MRI scanner (Magneton Vida, Siemens) and included gradient‐echo T2‐weighted, spin‐echo T1‐weighted, fat saturation fast spin‐echo and T2‐weighted sequences in coronal, sagittal and transverse orientations. The morphology and size of the scaffold were assessed according to the Genovese classification [8], while the morphology of the untreated contralateral meniscus was described according to Reicher et al. [15]. Subchondral bone reactions were evaluated using Lynch's criteria [12], and meniscus extrusion was measured post‐operatively using coronal views as described by De Coninck [6]. Extrusion was categorized as complete if the meniscal tissue overlapped the tibial plateau completely, and as partial if only partial overlap was present (Table S1). The MRI and radiographic assessment were independently evaluated by two surgeons specializing in sports medicine within a 48‐h timeframe.

### Statistical analysis

Mean, standard deviation (SD), minimum, and maximum values were calculated for all descriptive data. For continuous variables, the mean and 95% confidence intervals (CIs) were reported. Statistical analyses were performed using the paired *t*‐test for parametric data with a normal distribution. To assess arthritis progression, the Wilcoxon signed‐rank test was employed due to the ordinal nature of the data. A significance level of *p* < 0.05 was used to determine statistical significance. To assess the impact of concomitant procedures on clinical outcomes, we identified patients who underwent additional interventions including anterior cruciate ligament (ACL) reconstruction, ACL revision, microfracture, or treatment of a contralateral meniscal tear. Outcomes were compared between patients with and without concomitant procedures using independent two‐sample *t* tests assuming unequal variances (Welch's *t* test).

## RESULTS

### Patient demographics

The study population (Table 1) consisted of 30 consecutive patients (24 men and 6 women), with a mean age of 25 years upon implementation (ranging from 15 to 36 years, SD = ±6.7). Notably, two had bilateral knee operations, making the total number of knees operated on 32.

**Table 1:** Patient characteristics.

Characteristics	Number of patients (%)
Mean age (SD)	25 (6.7)
Male/female	24 (80)/6 (20)
Right/left	21 (65)/11 (35)
Medial/lateral meniscus	4 (12.5)/28 (87.5)
Chondral lesions (Grades III and IV)	12 (37.5)
Kellgren–Lawrence Stage I post‐op	15 (47)
Kellgren–Lawrence Stage II post‐op	10 (31)
Kellgren–Lawrence Stage III post‐op	7 (22)

Abbreviation: SD, standard deviation.

The mean follow‐up duration was 11.4 years, with a range of 10–16 years. No intraoperative complications were recorded, and all patients received the same surgical procedure for the implantation of the Actifit® scaffold by the same senior author, M.H.

Among these patients, 21 had operations on their right knee and 11 on their left knee. Of the 32 knees operated on, 28 had lateral meniscus deficiency while 4 had medial meniscus deficiency. All patients had undergone previous meniscectomies, and four had ACL reconstructions. Of the 32 knees, 30 had been operated on once, and 2 had undergone two prior surgeries.

Some of the patients required concomitant procedures; however, each patient underwent only one additional procedure. Eight patients underwent combined ACL reconstruction, while four required revision ACL reconstruction. Twelve patients received microfracture treatment for Type IV cartilage lesions in the femoral condyle. In all cases, the area of cartilage lesions was less than 2 cm^2^. All 12 knees included in the study presented with lateral femoral condyle chondral lesions. In five patients, the contralateral meniscus was also repaired. All patients completed the follow‐up, with no losses to follow‐up.

Τhe mean meniscal defect size was 43.4 mm (range: 30–58 mm, SD = 7.2). The fixation of the implant was achieved through a hybrid suture technique, with a mean of 5.2 sutures (ranging from 4 to 7, SD = 1), combining all‐inside (mean: 2.9, SD = 1.1) and inside‐out (mean: 2.6, SD = 1.2) sutures.

### Patient‐reported outcomes

The study demonstrated significant (*p* < 0.05) improvements in clinical outcomes (Table 2). The KOOS improved from a preoperative mean of 47.75 (SD: 16.88) to a final follow‐up mean of 78.62 (SD: 15.45). The Lysholm score improved from a preoperative mean of 46.62 (SD: 16.96) to a final follow‐up mean of 84.62 (SD: 13.03). The VAS for pain decreased significantly from a preoperative mean of 5.62 (SD: 2.54) to a final follow‐up mean of 2.12 (SD: 2.02), indicating a substantial reduction in pain levels. The Tegner Activity Scale improved from a preoperative mean of 1.8 to a final follow‐up mean of 4.1. Patient satisfaction was high, with a mean score of 9.2 (range: 8–10, SD = 0.7).

**Table 2:** Patient‐reported outcomes.

	Preoperatively mean (SD) [95% confidence interval]	Post‐operatively mean (SD) [95% confidence interval]	*p*	Mean change (Δ)	Cohen's *d* (effect size)
KOOS	47.7 (16.8) [41.8–53.5]	78.6 (15.4) [73.2–83.9]	*p* < 0.05	+30.9	1.96
Lysholm score	46.6 (16.9) [40.7–52.4]	84.6 (13) [80–89.1]	*p* < 0.05	+38.0	2.51
VAS score	5.6 (2.5) [4.7–6.4]	2.1 (2) [1.4–2.7]	*p* < 0.05	−3.5	1.56
Tegner activity score	1.8 (0.3) [1.6–1.9]	4.1 (0.5) [3.9–4.2]	*p* < 0.05	+2.3	5.05

Abbreviations: KOOS, Knee injury and Osteoarthritis Outcome Score; SD, standard deviation.

### Radiological outcomes

The radiological evaluation revealed varying degrees of osteoarthritic changes according to the K–L classification. Preoperatively, 18 knees were classified as Type 0, and 14 knees as Type I. At the final follow‐up, among the 32 knees evaluated, 15 were classified as Type I, indicating doubtful osteoarthritic changes. Ten knees were classified as Type II, reflecting mild changes and seven knees were classified as Type III, denoting moderate osteoarthritic changes. Therefore, there was a slight deterioration of radiological outcomes (*p*‐value < 0.05).

Progression of arthritis was observed in all 12 patients who had advanced cartilage lesions (Grade IV). More specifically, preoperatively, 2 of these knees were classified as Type 0, while 10 knees were classified as Type I. At the final follow‐up, seven of these knees were classified as Type III while the rest were classified as Type II (*p* value < 0.05). Notably, all 12 patients had arthritis localized to the lateral compartment.

### MRI evaluation

**Figure 1:**
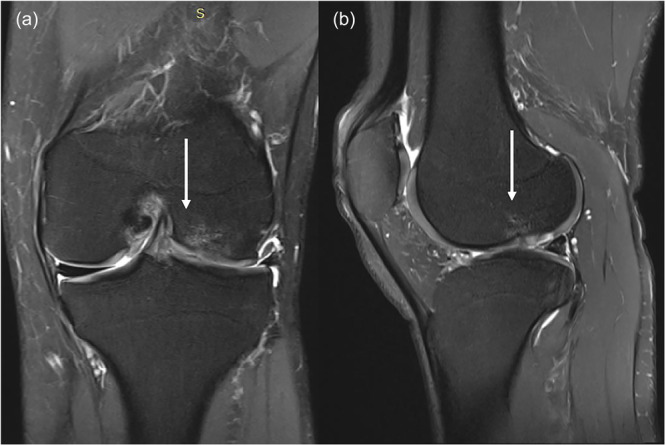
MRI of the left knee (a: coronal view—fat saturation, b: sagittal view—fat saturation) 12 years post‐operation on the lateral meniscus shows a maintained implant, though smaller in size, with partial extrusion. The white arrow indicates healed cartilage of the lateral femoral condyle following the microfracture technique, with evidence of subchondral oedema. MRI, magnetic resonance imaging.

**Figure 2:**
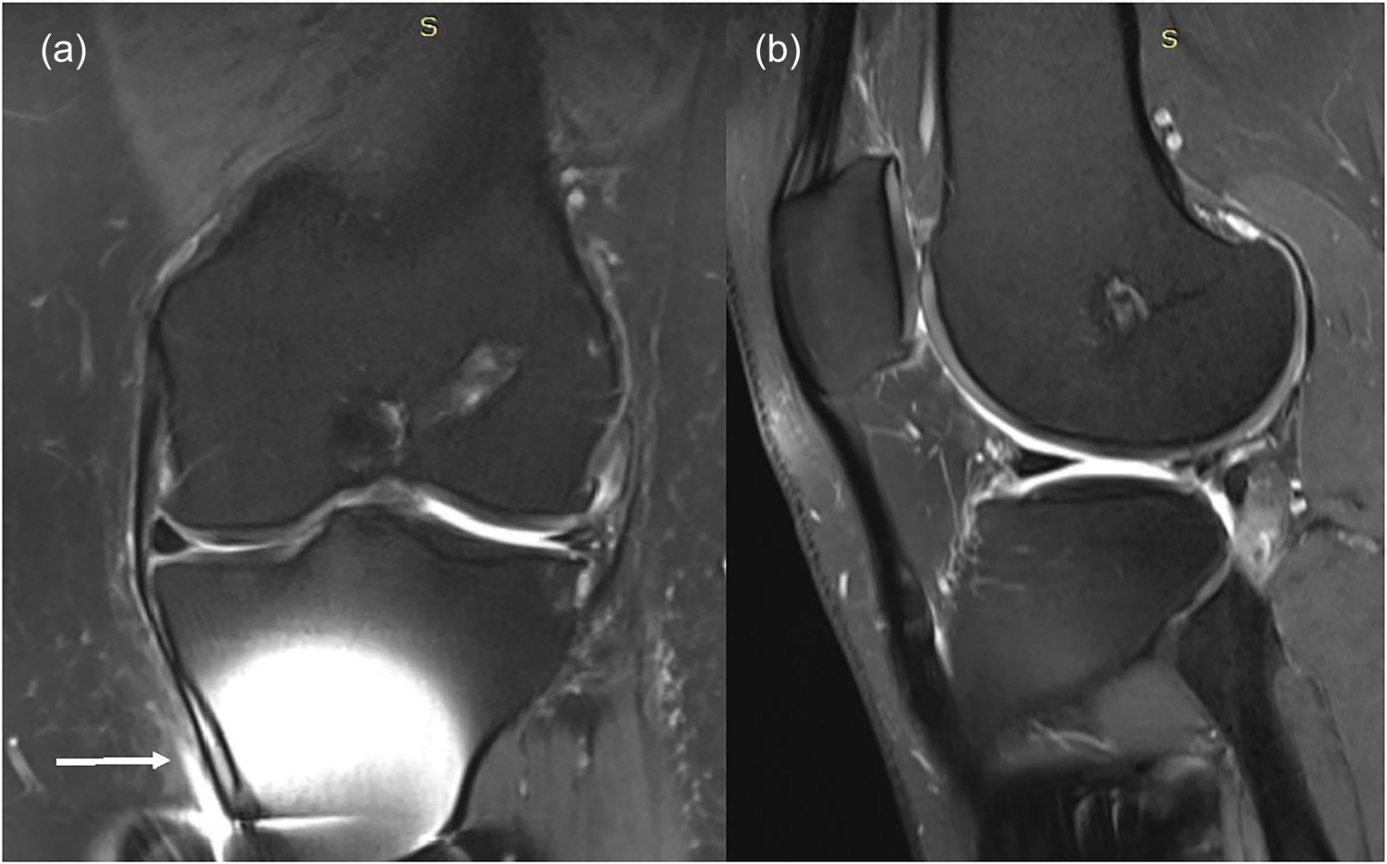
MRI of the left knee (a: coronal view—fat saturation, b: sagittal view—fat saturation) 11 years post‐operation on the lateral meniscus shows a maintained implant, though smaller in size, with partial extrusion and a tear. The white arrow indicates bone oedema, likely an artefact from ACL reconstruction. ACL, anterior cruciate ligament; MRI, magnetic resonance imaging.

According to the Genovese classification, the scaffold morphology and integration were categorized into three types. None of our patients were classified as Type III, showing scaffolds identical in size and shape to the normal meniscus. Twenty‐three knees (71.88%) were classified as Type II (Figure 1), indicating a smaller scaffold with either regular or irregular morphology, demonstrating that the scaffold had integrated but was smaller than the native meniscus. Nine knees (28.12%) were classified as Type I (Figure 2), indicating total resorption of the scaffold, suggesting a failure rate of 28.12% of the scaffold.

Bone oedema, indicative of subchondral bone stress or irritation, was not present in 27 knees, reflecting minimal to no subchondral stress. However, five knees exhibited Type I bone oedema, indicating some degree of subchondral bone irritation.

Meniscal extrusion was another critical factor assessed in the MRI evaluations. Partial extrusion of the scaffold was observed in 24 knees, while complete extrusion was seen in 8 knees. The condition of the contralateral meniscus was also assessed. In 28 patients, the contralateral meniscus was normal, while in 2 patients, it was graded as Reicher Grade 1, indicating minor degenerative changes.

Patients with concomitant procedures demonstrated significantly better post‐operative Lysholm scores (mean = 91.0, SD = 6.8) compared to those without (mean = 74.0, SD = 14.8; *p* = 0.002). They also reported significantly lower VAS scores (mean = 1.4, SD = 1.4 vs. 3.3, SD = 2.5; *p* = 0.025), indicating reduced pain levels. Differences in KOOS (*p* = 0.055), satisfaction (*p* = 0.436) and Tegner scores (*p* = 0.112) did not reach statistical significance.

## DISCUSSION

This study demonstrates that the Actifit® polyurethane meniscal implant provides favourable clinical outcomes and maintains low pain levels over a long‐term follow‐up period of at least 10 years. Approximately 72% of patients retained the implant's structure, although with a reduction in size, while complete resorption of the Actifit® substitute occurred in 28% of cases. Bone oedema in the affected condyle was observed in only 15% of patients, and 22% exhibited signs of moderate arthritis. Notably, all patients with moderate arthritis had Grade IV chondral lesions at the time of implantation, and their arthritis stage progressed significantly over the 10‐year follow‐up period. These findings support the study hypothesis. To our knowledge, this is the first study to report minimum 10‐year MRI results on Actifit® meniscal implantation.

Previous literature supports the short‐term benefits of the implant, with studies by Verdonk et al. [19], Bouyarmane et al. [2] and De Coninck et al. [6] showing significant improvement in clinical outcomes up to 24 months. Verdonk et al. [19] reported clinically and statistically significant improvements in all outcome scores at 2‐year follow‐up following Actifit® implantation, including marked reductions in pain (VAS: 45.7 to 20.3) and improvements in function (International Knee Documentation Committee [IKDC]: 45.4–70.1; Lysholm: 60.1–80.7; and KOOS subscales). Similarly, Bouyarmane et al. [2] found consistent improvement in VAS (5.5–2.9) and IKDC (47.0–67.0) over 24 months, with all KOOS domains showing notable gains. In comparison, the present study, despite having a significantly longer follow‐up of a minimum of 10 years, demonstrated sustained improvements: KOOS improved from 47.7 to 78.6, Lysholm from 46.6 to 84.6, and VAS from 5.6 to 2.1. Notably, the Tegner activity score also increased from 1.8 to 4.1. These long‐term outcomes not only confirm the short‐term benefits reported in earlier studies but also highlight the durability of clinical improvement over a decade. Bulgheroni et al. [3] reported that MRI evaluations at 2 years post‐implantation showed the presence of the scaffolds, although they exhibited differences in shape, size, and intensity compared to the native meniscus. Additionally, they observed no progression of degenerative processes in the knee joint, indicating a potential protective effect of the implant on the articular cartilage. Gelber et al. [7] recommended the use of Actifit® in patients without chondral lesions, as these individuals exhibited a more favourable MRI appearance of the polyurethane scaffold in terms of size and morphology. Consistent with these findings, our study further suggests that the presence of grade IV chondral lesions at the time of implantation is a significant factor, for progression to arthritis at the 10‐year follow‐up mark.

Baynat et al. [1] found polymer ingrowth by normal chondrocytes and fibrochondrocytes with histological examination and no damage to the implant a year after the implantation was performed, stating that the implant induces and promotes meniscal regeneration. Additionally, Schüttler et al. [16] reported mid‐term success, restoring knee joint function and significantly reducing pain in patients with segmental medial meniscus deficiency up to 4 years post‐implantation.

Similar findings were reported in studies with a minimum follow‐up of 5 years. Leroy et al. [10] followed 13 patients and observed abnormal MRI appearances, including intermediate signals and reduced implant size, indicating incomplete scaffold maturation. Despite this, functional scores and cartilage condition remained stable over time. Similarly, Monllau et al. [13] reported good functional outcomes at 5 years following Actifit® implantation, despite MRI evidence of reduced implant volume and abnormal morphology. Our findings are consistent, showing sustained improvements in KOOS, Lysholm, and VAS scores over a longer follow‐up of 10 years, even though 28% of patients had complete scaffold resorption and 72% exhibited structural changes on MRI. These results support the idea that favourable clinical outcomes can persist independently of scaffold MRI appearance.

In a multicentre study by Toanen et al. [17] involving 137 patients with a minimum follow‐up of 5 years, the polyurethane meniscal implant demonstrated the ability to improve knee joint function and reduce pain in patients with segmental meniscal deficiency. Despite these clinical improvements, MRI evaluations showed that the scaffold's appearance differed from the native meniscal tissue at midterm follow‐up. The treatment survival rates were 87.9% for medial scaffolds and 86.9% for lateral scaffolds.

de Chanterac et al. [5] evaluated 18 patients after Actifit® implantation and reported a 38% rate of full resorption of the implant after 8 years, along with significantly improved clinical outcomes. The average age of patients in their study was 34 years at the time of implantation. In contrast, our study demonstrated a lower rate of full resorption (28%) in a younger cohort of patients, with a mean age of 25 years. Furthermore, the clinical outcomes in our study, as measured by the Lysholm score, were higher (mean score: 84.6) compared to those reported by de Chanterac et al. (mean score: 79). Additionally, 33% of the patients in their study exhibited progression to arthritis, whereas only 22% of patients in our cohort showed similar progression.

In the study by Torres‐Claramunt et al. [18], 21 patients with a mean age of 56 years were followed for an average of 11.8 years after Actifit® implantation. They reported significant improvements in all functional scores (KOOS, IKDC and Lysholm), except for the Tegner score, which remained stable over time. While joint space width was maintained at 5 years, it significantly decreased by final follow‐up (from 1.9 to 0.6 mm, *p* = 0.001), reflecting radiographic progression of osteoarthritis. In comparison, our study, with a younger patient cohort (mean age 25 years), also demonstrated sustained functional improvements over 10 years, with marked gains in KOOS, Lysholm, and Tegner scores. However, unlike Torres‐Claramunt et al., we observed progression of arthritis primarily in patients with pre‐existing Grade IV chondral lesions, and the rate of K–L Grade III was 22%. Together, these findings support the long‐term clinical efficacy of the Actifit® scaffold while highlighting that degenerative progression may still occur, especially in older patients or those with advanced cartilage damage at baseline.

The study's findings indicate that the majority of patients did not demonstrate significant progression to arthritis over the long‐term follow‐up period, with the exception of those who presented with Grade IV chondral lesions at the time of implantation. These patients exhibited statistically significant advancement in their arthritis stages, highlighting the importance of the preoperative cartilage condition in determining long‐term outcomes. This observation underscores the need for careful patient selection when considering Actifit® implantation, as those with advanced cartilage damage may have a higher risk of degenerative progression despite the initial clinical improvements. To assess the clinical relevance of post‐operative improvements, we compared the observed changes in KOOS and Lysholm score to established minimum clinically important differences (MCIDs). While no MCID values are currently available specifically for synthetic graft implantation, we referenced Liu et al.'s [11] study on meniscal allograft transplantation, which provides a distribution‐based MCID of 12.3 for the Lysholm score. For the KOOS subcategories, Liu et al. reported MCIDs of 9.9 (Pain), 9.7 (Symptoms), 9.5 (ADL), 13.3 (Sports) and 14.6 (QoL). Using these, we derived an estimated overall KOOS MCID of 11.4 points to serve as a benchmark for clinical significance.

Patients in our study demonstrated substantial improvements that exceeded these thresholds. The KOOS increased from a preoperative mean of 47.7 (SD: 16.8) to 78.6 (SD: 15.4), representing a gain of 30.9 points, which is nearly three times the estimated MCID. Similarly, the Lysholm score improved from 46.6 (SD: 16.9) to 84.6 (SD: 13.0), a difference of 38.0 points, also far surpassing the MCID of 12.3. These findings indicate that the improvements following synthetic graft implantation were not only statistically significant but also clinically meaningful from the patient's perspective. Regarding concomitant procedures, the findings suggest that patients who undergo additional interventions alongside Actifit® implantation—such as ligament reconstruction or cartilage repair—may achieve comparable or even superior clinical outcomes in terms of function and pain relief. These procedures may enhance joint stability or the biological environment. Therefore, the presence of concomitant procedures should not be viewed as a negative prognostic factor; instead, they may contribute positively when appropriately indicated. In our centre, polyurethane scaffold implantation is indicated for young, active patients with symptomatic post‐meniscectomy syndrome, partial meniscal deficiency (with preserved horns and peripheral rim), neutral alignment, minimal degenerative changes (K–L Grade 0–I), and stable ligaments. These criteria aim to ensure optimal conditions for scaffold integration and long‐term success.

One notable finding in our study is the mismatch between clinical outcomes and MRI appearances in several cases. While the majority of patients reported significant and sustained functional improvement and pain reduction, imaging often revealed residual scaffold irregularity, reduction in size or even full resorption. This suggests that the biomechanical role or even partial presence of the scaffold may still offer functional benefit. This apparent mismatch may be explained by the scaffold's ability to provide temporary biomechanical support and promote fibrous tissue ingrowth, which can maintain joint function even as the material degrades. Additionally, the scaffold's mechanical properties differ from native meniscal tissue, making it more susceptible to micromotion and extrusion under load. Importantly, MRI findings may not always reflect clinical performance, as structural changes on imaging do not necessarily correlate with symptoms or poor clinical outcomes. These observations suggest that scaffold integrity on MRI should be interpreted cautiously and always considered alongside clinical assessment. Future research combining advanced biomechanical modelling with imaging and clinical data is needed to clarify the functional role of meniscal synthetic implants and guide post‐operative expectations.

Despite the positive outcomes observed in this study, several limitations should be noted. First, the retrospective nature of the study and the relatively small sample size may limit the generalizability of the findings. Second, the study population consisted of relatively young patients with a mean age of 25 years; thus, the findings may not be applicable to older populations or those with advanced degenerative changes. Third, the lack of a control group prevents direct comparisons with alternative treatments. The presence of concomitant procedures in several patients introduces a potential source of confounding, as these interventions—though often clinically necessary—may have independently contributed to the observed clinical improvements. While each patient underwent only one additional procedure, isolating the specific impact of the scaffold itself from the effects of these concomitant treatments remains challenging. Future prospective, controlled studies are warranted to more clearly define the independent role of the scaffold in clinical outcomes.

## CONCLUSIONS

The study demonstrated that arthroscopic treatment using a polyurethane meniscal implant for chronic segmental meniscal loss led to significant long‐term improvements in pain reduction and knee function. Despite radiographic evidence of osteoarthritic progression and the presence of extrusion, high levels of patient satisfaction were reported. These results suggest that the procedure provides sustainable long‐term benefits over a mean follow‐up period of 11.4 years.

## AUTHOR CONTRIBUTIONS

Study design, data collection, data analysis, manuscript drafting and critical revision of the manuscript: Vasileios S. Akrivos. MRI and radiological evaluation, Data analysis, statistical analysis, manuscript review and editing: George A. Komnos. MRI and radiological evaluation, interpretation of imaging data and manuscript review: Bela Ujvari. Patient follow‐up assessments, data collection and manuscript review: Artemis Hante. Patient recruitment, data collection and manuscript editing: Eleftheria Adaktylidou. Supervision, critical revision of the manuscript for important intellectual content and final approval of the manuscript: Theofilos Karachalios. Principal investigator, study conception and design, surgical procedures, critical revision of the manuscript, and final approval: Michael Hantes.

## CONFLICT OF INTEREST STATEMENT

The authors declare no conflicts of interest.

## ETHICS STATEMENT

The study was approved by the Scientific Committee of the University General Hospital of Larisa (approval number: 2957). All participants provided written informed consent prior to their inclusion in the study.

## Supporting information

Supporting information.

## Data Availability

The data sets generated and analyzed during the current study are not publicly available due to patient confidentiality and institutional policies but are available from the corresponding author upon reasonable request.
