# Supplementary material for: Long‐term clinical and MRI outcomes of a polyurethane meniscal scaffold implantation for the treatment of partial meniscal deficiency: A minimum 10‐year follow‐up study
Source: Knee Surg Sports Traumatol Arthrosc. 2025 Jun 15;34(5):1597–605. doi: 10.1002/ksa.12724 (PMC13122752; doi:10.1002/ksa.12724)
Supplement: Supplementary file 1 — Supporting information. [file KSA-34-1597-s001.docx]

| Classification System | Grading Categories | Definition / Description |
| --- | --- | --- |
| Genovese Classification (Scaffold Morphology – MRI) | Type I | Total resorption of the scaffold. No remaining implant visible. |
|  | Type II | Reduced size scaffold with regular or irregular morphology, indicating partial integration. |
|  | Type III | Scaffold identical in size and shape to the native meniscus. |
| Reicher Classification (Contralateral Meniscus – MRI) | Grade 1 | Homogeneously black meniscus(No tear) |
|  | Grade 2 | Region of very minimally increased signal intensity within the meniscus, usually not present on two adjacent scans (tear unlikely) |
|  | Grade 3 | Small, linear region of increased signal intensity or, alternatively, a small-to- moderate nonlinear area of increased signal intensity within the meniscus (probable tear) |
|  | Grade 4 | Gross distortion of the normal shape, truncation of the meniscus, or a large focus or line of increased signal intensity within the meniscus (definite tear). |
| Lynch Classification (Subchondral Bone Edema – MRI) | Type I | Loss of signal intensity that was primarily located within the medullary space of the bone, usually involving both the epiphyseal and metaphyseal regions without an interruption of the cortex |
|  | Type II | Loss of signal intensity that was associated with an interruption of the black cortical line |
|  | Type III | Loss signal intensity that was primarily restricted to the region of the bone immediately adjacent to the cortex without a definite cortical interruption |

Supplemental Table 1: MRI Classification Systems
